# Clinical Characteristics and Survival of Patients with Idiopathic Pulmonary Fibrosis: Analysis of the Serbian Cohort from the EMPIRE Registry

**DOI:** 10.3390/diagnostics15172121

**Published:** 2025-08-22

**Authors:** Sanja Dimic-Janjic, Mihailo Stjepanovic, Slobodan Belic, Dragan Vukosavljevic, Ivan Milivojevic, Nikola Trboljevac, Nikola Nikolic, Slavko Stamenic, Maja Stojanovic, Kristina Stosic, Martina Koziar Vasakova, Ruza Stevic, Nikola Colic, Katarina Lukic, Miroslav Ilic, Lidija Isovic, Nikola Maric, Spasoje Popevic, Violeta Vucinic-Mihailović, Svetlana Kasikovic Lecic, Slavica Mojsilovic, Tatjana Pejcic, Dragana Jovanovic, the Serbian EMPIRE Investigators

**Affiliations:** 1 Faculty of Medicine, University of Belgrade, 11000 Belgrade, Serbia; 2 Clinic for Pulmonology, University Clinical Center of Serbia, 11000 Belgrade, Serbia; 3 Clinic for Allergology and Immunology, University Clinical Center of Serbia, 11000 Belgrade, Serbia; 4 Department of Respiratory Medicine, Thomayer University Hospital, First Faculty of Medicine, Charles University Prague, 12108 Prague, Czech Republic; 5 Center for Radiology, University Clinical Center of Serbia, 11000 Belgrade, Serbia; 6 Faculty of Medicine, University of Novi Sad, 21000 Novi Sad, Serbia; 7 Institute for Pulmonary Diseases of Vojvodina, 21000 Novi Sad, Serbia; 8 Clinic for Pulmonology, University Clinical Center of Kragujevac, 34000 Kragujevac, Serbia; 9 Faculty of Medicine, University of Nis, 18000 Nis, Serbia; 10 Clinic for Pulmonology, University Clinical Center of Nis, 18000 Nis, Serbia

**Keywords:** idiopathic pulmonary fibrosis, forced vital capacity, diffusion capacity for carbon monoxide, usual interstitial pneumonia, antifibrotic treatment

## Abstract

**Background/Objectives**: Idiopathic pulmonary fibrosis (IPF) registries are established to enhance understanding of its natural history. **Methods**: Serbia (RS) participated in the EMPIRE (European Multi-Partner IPF Registry) from June 2015 to October 2022, involving four centers. The registry included patients over 18 diagnosed with IPF based on the 2011 international criteria. We aimed to gather key clinical, functional, and survival data, along with treatment information for IPF patients in RS, using a centralized electronic case report for consistency. **Results**: 188 RS patients participated (median age at diagnosis 65, 63.8% male, 51% smoking history, 56% radiological usual interstitial pneumonia (UIP) pattern). At the diagnosis, median forced vital capacity (FVC) was 73.7% and diffusion capacity for carbon monoxide (DL_CO_) was 38%. At initiation of antifibrotic therapy, median FVC was 73.2% (71.5% for deceased, 75.8% for survivors (*p* = 0.455), and DL_CO_ was 33.8% (19.9% for deceased, and 35.6% for survivors (*p* = 0.046)). The median long-term survival from diagnosis was 29.4 months (95% CI: 22.6–36.2 months), and 9.4 months (95% CI: 5.9–12.9 months) from the initiation of therapy, with no difference in the duration of antifibrotic treatment between survivors and deceased (*p* = 0.598). **Conclusions**: The RS EMPIRE cohort represents a younger, less comorbid population with fewer smokers and more probable UIP, factors linked to a favorable prognosis. Nevertheless, survival was poorer than expected, mainly due to advanced disease severity at the time of antifibrotic initiation, as indicated by lower DL_CO_. These findings highlight the importance of earlier diagnosis and treatment before significant physiological decline to improve outcomes.

## 1. Introduction

Idiopathic pulmonary fibrosis (IPF) is a chronic interstitial lung disease characterized by progressive pulmonary parenchymal damage and a decline in lung function. The etiology of IPF remains idiopathic; however, it is hypothesized to arise from an interplay of genetic predisposition, abnormal immune responses, and environmental exposures [1]. Genetically susceptible individuals have either mutations in genes related to epithelial cell function and telomere maintenance, mutations in surfactant protein, or specific single-nucleotide polymorphisms [2]. Despite advancements in comprehending the pathophysiology of IPF, the underlying mechanisms are believed to involve recurrent epithelial injury, epithelial dysfunction, abnormal repair leading to lung remodeling, and the development of fibrosis [3,4]. IPF is characterized by dysregulated interaction between epithelial cells and stromal cells, which are crucial sources of extracellular matrix (ECM) proteins [5,6]. Newer studies have demonstrated that an elevation in the number of stromal cells, including fibroblasts and endothelial, mural, and epithelial cells, as well as interactions between epithelial cells (ECs) and fibroblasts, particularly several identified key subtypes, may play a role in the development and progression of IPF [7]. Clinical manifestations of IPF commonly include persistent cough, fatigue, and progressive exertional dyspnea. This condition predominantly affects older adults, particularly individuals in their sixties, with an untreated average survival rate of approximately 3–5 years post-diagnosis [8]. Diagnosis is contingent upon clinical symptomatology, high-resolution computed tomography (HRCT), and pulmonary function testing. In atypical cases, cryobiopsy and surgical lung biopsy are utilized to establish a definitive diagnosis. The differential diagnosis is essential, as IPF shares overlapping features with various other interstitial lung diseases [9]. Currently, there is no definitive cure for IPF; however, advancements in pharmacotherapy, particularly the use of antifibrotic agents, aim to mitigate symptoms and slow disease progression, thereby enhancing patients’ quality of life [10,11]. This research was guided by the hypothesis that assessing the clinical, functional, and imaging characteristics of IPF patients would enable the identification of potential prognostic biomarkers and therapeutic targets to enhance patient management.

## 2. Materials and Methods

The EMPIRE (European Multi-Partner IPF Registry) project was established in 2014 as a non-interventional, international multicenter database for patients with IPF in Central and Eastern Europe. The Republic of Serbia (RS) is one of eleven countries included in the registry with four participating centers (Clinic for Pulmonology, University Clinical Center of Serbia; Clinic for Pulmonology, University Clinical Center of Kragujevac; Clinic for Pulmonology, University Clinical Center of Nis; and Institute for Pulmonary Diseases of Vojvodina). The primary objectives of the registry are to assess the incidence, prevalence, and mortality of IPF in this region, as well as to identify the fundamental characteristics of patients with IPF. Additionally, the registry aims to gather valuable information regarding the treatment of IPF patients within the specified area. Data from the EMPIRE project is stored in a highly secure online database system. The online registry application is accessible to users via an internet browser. Data entry is performed using electronic case report forms by qualified personnel. Each patient’s identity is replaced with a unique number (ID) known only to the attending physician. Intermittent data reviews and crosschecks are performed, and if any errors or discrepancies are identified, the site is notified of the corrections. Centralized administration and uniform forms for data collection at all sites facilitate the synthesis of the data and comparability between sites. Although formal inter-observer variability analysis is not performed, all participating centers followed a pre-specified standardized imaging and functional assessment protocol.

### 2.1. Study Population, Inclusion Criteria, Baseline, and Follow-Up Assessment

To be eligible for participation in the registry, patients must be at least 18 years old with a diagnosis of IPF based on the diagnostic criteria outlined in the 2011 international guidelines by the European Respiratory Society, the American Thoracic Society, the Japanese Respiratory Society, and the Latin American Thoracic Association [12], as evaluated by the treating physician, and confirmed by a multidisciplinary team during a face-to-face discussion. The EMPIRE registry was initiated in September 2014, with follow-up visits every 3–6 months. Serbian cohort participants, both incident and prevalent, were included in the EMPIRE registry from June 2015 to October 2022. Patients are followed in the registry until death or lung transplantation. The baseline analysis included demographics, smoking history, diagnostic evaluation of IPF, symptoms, risk factors, comorbidities, previous and current medications, lung function tests (such as forced vital capacity [FVC], forced expiratory volume in 1 s [FEV1], and diffusing capacity of the lungs for carbon monoxide [DL_CO_]), 6 min walk test (6MWT), high-resolution computed tomography (HRCT) patterns, and other variables. Follow-up assessments covered symptoms, acute exacerbations, lung function tests, disease management and treatment, resource utilization, outcomes, adverse events, quality of life, and treatment options, including pharmaceuticals, rehabilitation, oxygen therapy, lung transplantation, and mortality.

### 2.2. Pulmonary Function Tests and Imaging

The functional assessments were conducted according to the American Thoracic Society/European Respiratory Society (ATS/ERS) criteria [13,14]. Post-bronchodilator spirometry was performed using the JAEGER^®^ MasterScreen Pneumo (Vyaire Medica GmbH, Hochberg, Germany) and single-breath diffusion capacity for carbon monoxide (CO) was performed using the JAEGER^®^ MasterScreen Diffusion (Vyaire Medica GmbH, Hochberg, Germany). HRCT was performed on a 128-slice CT scanner, Siemens Somatom DefinitiON Edge (Siemens Healthineers, Forchheim, Germany), in native phase with a care dose option (approximately 120 kV, 110 mAs), a rotation time of 0.7 s, and a speed of 42 mm/s. Reconstructions included a lung kernel (no. 46, 0.6 mm). 6MWDT was performed by the ATS guidelines [15].

### 2.3. Statistical Analysis

Numeric data were summarized using either the mean and standard deviation or the median and range, depending on the normality of the distribution. Normality was assessed through the Shapiro–Wilk test and box plots. Categorical data were described using absolute counts and relative percentages. To compare different groups based on numerical variables, Student’s *t*-test or the Mann–Whitney test was used, depending on the distribution’s normality. For categorical data, the Chi-square test was applied. The association between parameters was evaluated using Spearman’s rank correlation coefficient. The Kaplan–Meier method was used to estimate survival over time from diagnosis until death from any cause. Univariate and multivariate logistic regression analyses were conducted using the enter method to identify factors associated with mortality. Odds ratios (OR), 95% confidence intervals (95% CI), and *p*-values were reported. All statistical analyses were considered significant at a confidence level of 0.05. The study was performed using IBM SPSS Statistic for Windows, version 26.0 (Armonk, NY, USA: IBM Corp., 2019).

## 3. Results

### 3.1. Regional Subject Distribution

A total of 188 patients from RS were included in the EMPIRE registry. Data were collected from four university clinical centers in Serbia: Clinic for Pulmonology at the University Clinical Centre of Serbia in Belgrade, (*n* = 93; 49.5%); Institute for pulmonary diseases of Vojvodina (*n* = 72; 38,3%); Clinic for Pulmonology, University Clinical Center of Kragujevac (*n* = 12; 6.4%); Clinic for Pulmonology, University Clinical Center of Nis (*n* = 11; 5,8%).

### 3.2. Follow-Up

At the time of data analysis, 57 of the subjects (30%) had died, while only 1 subject (1%) had undergone lung transplantation. Additionally, 24 (13%) were lost to follow-up, and 6 (3%) had their diagnosis changed. Another 2 subjects (1%) were censored for unspecified reasons, leaving 98 (52%) of the patients alive at the time of analysis. Most patients were followed for less than 12 months (45.2%), with a small percentage (7.3%) followed for more than 108 months. For those whose follow-up was terminated, the median duration was 14.2 (3–31.1) months, with an average follow-up duration of 27 months. Among those still under follow-up, the median duration was 15.2 (6.4–40.0) months, with an average of 40.2 months.

### 3.3. Baseline Characteristic

Baseline characteristics of the 188 included patients are presented in Table 1, showing that the majority were newly diagnosed, predominantly male, with a median age at diagnosis of 65 years and a median symptom duration of 12 months.

### 3.4. Radiological Pattern

The most common radiological pattern at the time of enrollment was usual interstitial pneumonia (UIP) in 56% of subjects (*n* = 79), followed by 35% with a probable UIP pattern (*n* = 50), and 9% inconsistent with UIP (*n* = 13). When performed (9%; *n* = 14), histopathological confirmation was consistent with probable UIP.

### 3.5. Comorbidities

Regarding comorbidities recorded at any time in the registry, 42% of patients had at least one comorbidity, and 33% had at least two. Three comorbidities were found in 5.9% of patients. The most common comorbidity type was cardiovascular (69.7%), followed by gastrointestinal and metabolic comorbidities (25%), while pulmonary comorbidities were present in 11.7% of patients. The most common disease-specific comorbidities included arterial hypertension (51.6%), diabetes mellitus (DM) (12.2%), and coronary artery disease (CAD) (8.5%).

### 3.6. Therapy

Among the 188 patients in the registry, pharmacotherapy was utilized by 89.1% (*n* = 168), oxygen therapy was administered to 28.4% (*n* = 53), and rehabilitation was employed for 16.5% (*n* = 31). Among those receiving pharmacotherapy, 62.5% (*n* = 115) were treated with Pirfenidone or Nintedanib (treatment switch included), 39.1% (*n* = 72) with Pirfenidone, 39.7% (*n* = 73) were treated with systemic corticosteroids, 28.3% (*n* = 52) with Nintedanib, 17.9% (*n* = 33) with N-acetylcysteine, 12.5% (*n* = 23) with Azathioprine, 7.1% (*n* = 13) were treated with proton pump inhibitors, and 9.2% (*n* = 17) with other cytostatic/immunosuppressive drugs. There was no statistically significant difference in the duration of antifibrotic therapy between patients who died and those who did not, according to the Mann–Whitney U test (*p* = 0.598) (Table 2).

### 3.7. Pulmonary Function Tests

Lung function parameters (FVC, FEV_1_, DL_CO_) at the time of diagnosis (Table 1) and at initiation of therapy were assessed (Table 3). DLco at the time of the initiation of antifibrotic treatment was significantly lower among patients who died than among those who survived (*p* = 0.046) (Table 3).

### 3.8. Survival

**Figure 1:**
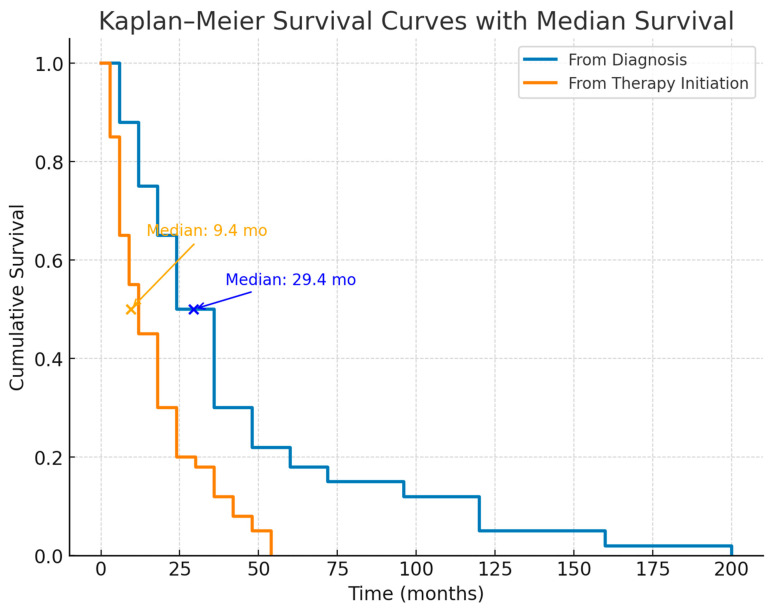
The Kaplan–Meier survival curves with median survival in patients with idiopathic pulmonary fibrosis (IPF).

**Figure 2:**
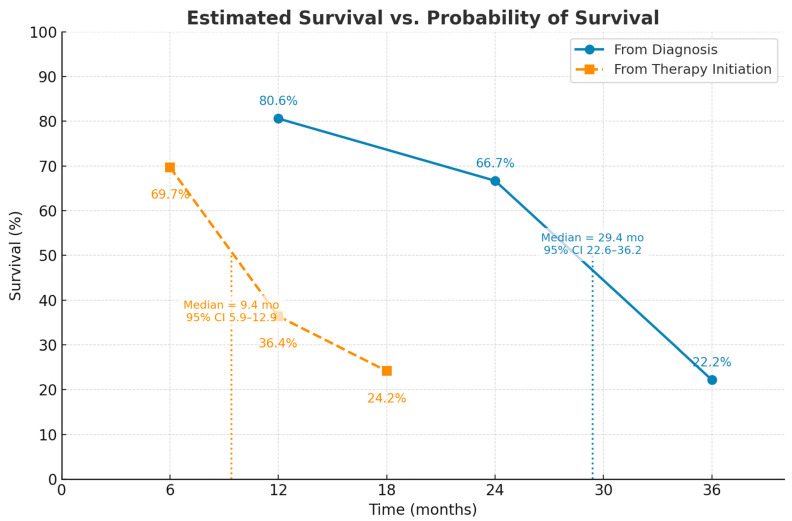
Kaplan–Meier estimates of survival in patients with idiopathic pulmonary fibrosis (IPF). Estimated survival rates from the time of diagnosis are shown in blue (solid line). In contrast, the probability of survival from the time of antifibrotic therapy initiation is shown in orange (dashed line). Median overall survival from diagnosis was 29.4 months (95% CI, 22.6–36.2), compared with 9.4 months (95% CI, 5.9–12.9) from therapy initiation. Data points indicate survival probabilities at fixed time intervals, with exact percentages annotated. Vertical dotted lines mark median survival estimates. The figure highlights the substantially reduced survival once antifibrotic therapy was initiated, reflecting advanced disease stage at treatment onset.

The median overall survival from the time of diagnosis was 29.4 months (95% CI, 22.6–36.2) (Figure 1). Estimated survival rates were 80.6% at 12 months, 66.7% at 24 months, and 22.2% at 36 months (Figure 2). Among the 188 patients included in the analysis, the median survival from initiation of therapy was 9.4 months (95% CI, 5.9–12.9) (Figure 1). The probability of survival at 6, 12, and 18 months following treatment initiation was 69.7%, 36.4%, and 24.2%, respectively (Figure 2).

The figure demonstrates a marked difference in survival when measured from diagnosis versus from therapy initiation. Median survival from diagnosis was approximately 29.4 months, whereas median survival from therapy initiation was only about 9.4 months. The steeper early decline in the post-therapy curve suggests that many patients began treatment at an advanced disease stage, when mortality risk was already high. In contrast, survival from diagnosis shows a longer initial plateau, reflecting a subset of patients with slower disease progression prior to therapy.

## 4. Discussion

IPF registries reveal real-world, longitudinal data on natural history, clinical events, patient-reported outcomes, quality of life, practice patterns, and treatment effectiveness. Participants from our EMPIRE-RS IPF cohort have a wide range of ages, clinical aspects, disease severity, and comorbidities, which is characteristic of real-world IPF compared to clinical trial cohorts. We demonstrated markedly different survival patterns when measured from diagnosis versus from the initiation of antifibrotic therapy, with median overall survival of 29.4 months and 9.4 months, respectively. The more rapid decline after therapy initiation indicates that many patients began treatment at a late stage of disease. Supporting this, diffusion capacity of the lungs (DL_CO_) at the time of antifibrotic therapy initiation was significantly lower in patients who subsequently died compared to those who survived (*p* ≈ 0.046), consistent with more advanced functional impairment in the non-survivor group. In contrast, there was no statistically significant difference in the duration of antifibrotic therapy between survivors and non-survivors. This suggests that poorer outcomes in the deceased group were not due to shorter treatment exposure, but likely related to worse baseline disease severity at therapy initiation. Together, these findings underscore the prognostic importance of functional status when starting antifibrotic therapy and suggest that earlier initiation—before marked DL_CO_ decline—may improve survival outcomes.

Comparing data from the EMPIRE-RS registry with other countries, we observe a different spectrum of registered patients. Bulgaria had the lowest number of recorded cases (*n* = 3), while the Czech Republic had the highest (*n* = 683) [16]. The median age of patients in EMPIRE-RS was 65 (min. 40–max. 85), in the Australian IPF Registry (AIPFR) mean 70.9 [17], EMPIRE 67.6 (with the oldest Austrian cohort 74) [18], INSIGT–IPF (Investigating Significant Health Trends in Idiopathic Pulmonary Fibrosis) 69.8 [19], Finnish 73 [20], Swedish 72.7 [21], and the IPF_PRO USA Registry 70 [22]. This makes our cohort one of the youngest, and this also aligns with findings from various studies that show the disease typically occurs between ages 50 and 70, with clinical symptoms often appearing after age 60 (12). Representation of ever-smokers in the EMPIRE-RS cohort was among the lowest in the EMPIRE registry (51%), in the overall registry 62.9%, while EMPIRE-Croatia had the highest percentage, 84.1%. Ever-smokers in Finland were 55%, the IPF_PRO USA Registry 68.4%, and INSIGT–IPF 63.3%. Most of the RS subjects were male, consistent with earlier research across all IPF registries. The BMI for RS subjects was the lowest among the compared registries at a mean of 26.8 kg/m^2^.

Considering the pulmonary function tests of the EMPIRE-RS cohort, the median FVC at diagnosis was 73.7% (min. 39%–max 120%), DL_CO_ was 38.1% (min. 11%–max. 79%), which is worse than the median EMPIRE (FVC 76.2%, DL_CO_ 47%) [23], but with higher FVC, and lower DL_CO_ than in INSIGT-IPF (mean FVC 68.6%, D_LCO_ was 37.8%) and IPF-PRO USA Registry (Median FVC 69.7% and DL_CO_ 40.6%). FVC is the most commonly used physiological parameter for the follow-up of IPF patients, as it is directly related to disease outcomes [24]. DL_CO_ is the product of how much effective surface is available (alveolar volume-VA) and how efficiently each unit of that surface transfers gas (transfer coefficient-K_CO_) [25]. As VA essentially reflects total lung capacity, unless there is an intrapulmonary airflow restriction, a natural correlation exists between DL_CO_ and FVC. A decrease in FVC generally accompanies a decline in DL_CO_. However, some patients may exhibit a drop in DL_CO_ without a significant change in FVC [26]. The association between DL_CO_ decline and mortality risk remains consistent even among patients with stable FVC. This may be due to the influence of various factors, such as microvascular damage. Given the individual heterogeneity of physiological impairments in the lungs of IPF patients, a decline in DL_CO_ may reflect different aspects of disease progression that are not detectable through changes in FVC. Therefore, serial measurements of both FVC and DL_CO_ should be conducted to monitor patients with IPF.

The most common radiological pattern at the time of enrollment was definite UIP in 79 patients (56%) of subjects (*n* = 79), followed by a probable UIP pattern in 35% of subjects (*n* = 50). Serbia had one of the lowest percentages of patients with a “typical” pattern of UIP—56%, also lower than the typical definite UIP in the whole EMPIRE registry (1550 patients (75.5%), atypical (possible or inconsistent with UIP) in 491 (24.5%)). Poland recorded the highest percentage (77.2%). In 2020, Fukihara et al. published a study comparing patients with definite and probable UIP patterns. They observed that the median survival for patients with a probable UIP pattern was significantly higher at 67.4 months compared to 42.6 months in those with a definite UIP pattern [24]. Notably, the time to first exacerbation and the annual decline in FVC were similar in both groups. The INPULSIS study, which demonstrated the benefit of nintedanib in treating IPF, also showed that patients with probable UIP had a higher median survival and a more extended period before their first exacerbation [27].

The context of IPF management practices is also reflected in the registry, probably due to changes in guidelines and treatment availability. Since it was a previous conventional treatment approach, a significant proportion of patients received oral corticosteroids (39.7%) and other immunosuppressive medications (16.3%), with later evidence indicating against the use of this combination in patients with IPF. Antifibrotics were used in 62.5% of cases, probably because the first antifibrotic was approved in Serbia in 2016, a year after the registry initiation, which is still higher than in Poland and Bulgaria. Austria reported the highest proportion of patients treated with antifibrotics, 78.2% (*n* = 55), followed by the Czech Republic with 72.5% (*n* = 971). Our findings also imply that earlier initiation of antifibrotic therapy before significant disease progression may improve survival in IPF, as shown by the much shorter survival when treatment began late. These findings are consistent with prior evidence that earlier initiation of antifibrotic treatment following IPF diagnosis is advantageous [28,29].

At the time of enrollment in the EMPIRE registry, most patients (91.3%) had at least one comorbidity, 56.4% had at least three, and more than one-third (37.8%) had at least four comorbidities. Most patients (73.9%) also had a cardiovascular type of comorbidity [30]. Additionally, the presence of multiple comorbidities at enrollment was linked to significantly worse survival. The RS EMPIRE cohort had the lowest comorbidity rate compared to other countries in the registry. It is important to note that our country’s patients had the lowest average age and the smallest percentage of smokers. In the RS cohort, 42% of patients had at least one comorbidity, with nearly 70% having cardiovascular comorbidity—the most common comorbidity in Serbia, likely due to regional lifestyle rather than IPF itself. Pulmonary hypertension was the only comorbidity likely influenced by interstitial lung disease, and according to Collum et al. [31], approximately 30% of patients with advanced IPF have diagnosed pulmonary hypertension. In a retrospective study, the median survival for IPF patients was 23 months, whereas for those with both IPF and pulmonary hypertension, it was only 11 months [32]. This may also influence the observed mortality trends in Serbia.

We previously emphasized that our findings support that starting antifibrotic therapy earlier, before marked DLCO decline, can improve survival in IPF, as worse outcomes are linked to late initiation rather than treatment duration. Serbian IPF patients, according to EMPIRE data, generally have poorer survival compared to Western European cohorts (Australia, Sweden, Germany, Finland, and the US IPF-PRO cohort) concerning 1-, 3-, and 5-year survival and median survival. This emphasizes the need for earlier diagnosis and broader antifibrotic use in Serbia to improve outcomes.

## 5. Conclusions

The RS EMPIRE cohort represents one of the youngest and least comorbid populations within the registry, characterized by the lowest smoking rates and the smallest proportion of patients exhibiting a definitive UIP pattern on HRCT—factors all associated with a more favorable prognosis. Additionally, the higher prevalence of probable UIP may be linked to better survival outcomes. Nevertheless, the cohort demonstrates relatively poorer survival compared to expectations based on these favorable characteristics, suggesting that other adverse factors—such as delayed diagnosis, limited access to advanced therapies, or barriers within the healthcare system—continue to influence patient outcomes negatively. In line with our previous findings, our results also underscore that poorer survival among patients with IPF was primarily related to advanced disease severity at the time of antifibrotic therapy initiation, as reflected by lower DL_CO_, rather than the duration of treatment exposure. These results emphasize the prognostic significance of functional status and suggest that earlier initiation of antifibrotic therapy, before substantial physiological decline, may improve survival outcomes. Earlier recognition of IPF facilitated by a streamlined referral pathway and timely access to functional diagnostics and HRCT, and more timely access to antifibrotic therapy are essential to improve survival in Serbia. Strengthening registry data and regional collaboration within EMPIRE could support better prognostic insight and help reduce disparities with other European cohorts.

## Figures and Tables

**Table 1:** Baseline characteristics of patients (*n* = 188).

Characteristic	Value	%/IQR/sd
Patient status	Newly diagnosed	162 (86.2)
	Diagnosed before database implementation	26 (13.8)
Gender	Male	63.8
	Female	36.2
Median age at diagnosis (years)	65	(60–72.2)
Median duration of symptoms	12 months	
<6 months		23.1
6–11 months		22.0
12–17 months		22.0
18–23 months		4.9
24–29 months		9.9
30–35 months		0.0
36–41 months		6.6
≥42 months		11.5
BMI (kg/m^2^)	Median	26.8
<20		3.2
20–24		33.5
25–29		41.1
30–34		16.8
35–39		4.9
Smoking status	Ever-smoker	51.0
	Nonsmoker	49.0
NYHA class	I	8.0
	II	59.0
	III	27.0
	IV	5.0
Main signs/symptoms	Dyspnea	91 (*n* = 169)
	Cough	58 (*n* = 107)
	Crepitation	92 (*n* = 171)
Lung function (% predicted)	FVC	73.7 (62.4–90.9)
	FEV_1_	79.8 (66.4–91.4)
	DLco	38.0 (26.9–49.3)

Abbreviations: FVC—forced vital capacity; FEV_1_—forced expiratory volume in one second; DLco—diffusing capacity for carbon monoxide; IQR—interquartile range, BMI—body mass index, NYHA—New York Heart Association.

**Table 2:** Duration of antifibrotic therapy.

Duration of Antifibrotic Therapy (Pirfenidone and Nintedanib) in Months	Median (IQR)	*p*
For all patients	10.6 (3.7–21.1)	0.598
Deceased	10.7 (5.3–20.4)	
Alive	10.5 (2.3–21.1)	

**Table 3:** Lung function parameters at initiation of therapy according to survival status.

Parameter	Overall Median (IQR)	Alive Median (IQR)	Deceased Median (IQR)	*p* Value
FVC (% predicted)	73.2 (62.7–88.3)	75.8 (65.7–88.1)	71.5 (53.9–92.9)	0.455
FEV_1_ (% predicted)	79.8 (67.2–93.1)	79.3 (69.9–96.4)	78.4 (53.9–93.1)	0.307
DLco (% predicted)	33.8 (17.2–46.8)	35.6 (24.3–47.1)	19.9 (unmeasurable–37.5)	0.046

Abbreviations: FVC—forced vital capacity; FEV_1_—forced expiratory volume in one second; DLco—diffusing capacity for carbon monoxide; IQR—interquartile range.

## Data Availability

The dataset is available on request from the authors.

## Abbreviations

The following abbreviations are used in this manuscript:

IPF	Idiopathic pulmonary fibrosis
FVC	forced vital capacity
FEV_1_	forced expiratory volume in one second
DLco	diffusion capacity for carbon monoxide
6MWDT	six-minute walk distance test
HRCT	high-resolution computed tomography
UIP	usual interstitial pneumonia
BMI	Body mass index.
